# Nutraceutical with Resveratrol and Omega-3 Fatty Acids Induces Autophagy in ARPE-19 Cells

**DOI:** 10.3390/nu8050284

**Published:** 2016-05-11

**Authors:** Ali Koskela, Mika Reinisalo, Goran Petrovski, Debasish Sinha, Céline Olmiere, Reijo Karjalainen, Kai Kaarniranta

**Affiliations:** 1 Department of Ophthalmology, University of Eastern Finland, Kuopio 70211, Finland; kai.kaarniranta@uef.fi; 2 School of Pharmacy, University of Eastern Finland, Kuopio 70211, Finland; mika.reinisalo@uef.fi; 3 Stem Cells and Eye Research Laboratory, Department of Ophthalmology, Faculty of Medicine, University of Szeged, Szeged 6720, Hungary; 4 Centre of Eye Research, Department of Ophthalmology, Oslo University Hospital, University of Oslo, Oslo 0450, Norway; goran.petrovski@medisin.uio.no; 5 Wilmer Eye Institute, Johns Hopkins University School of Medicine, Baltimore, MD 21287, USA; debasish@jhmi.edu; 6 Laboratoires Théa, Clermond-Ferrand cedex 63 000, France; c.olmiere@laboratoires-thea.fr; 7 Department of Biology, University of Eastern Finland, Kuopio 70211, Finland; reijo.karjalainen@uef.fi; 8 Department of Ophthalmology, Kuopio University Hospital, Kuopio 70029, Finland

**Keywords:** autophagy, p62/SQSTM1, LC3, proteasome, resveratrol, omega-3

## Abstract

Impaired autophagic and proteasomal cleansing have been documented in aged retinal pigment epithelial (RPE) cells and age-related macular degeneration (AMD). Omega-3 fatty acids and resveratrol have many positive homeostatic effects in RPE cells. In this work, ARPE-19 cells were treated with 288 ng of Resvega, containing 30 mg of trans resveratrol and 665 mg of omega-3 fatty acids, among other nutrients, with proteasome inhibitor MG-132 or autophagy inhibitor bafilomycin A1 up to 48 h. Autophagy markers p62/SQSTM1 (p62) and LC3 (microtubule-associated protein 1A/1B-light chain 3) were analyzed by Western blotting. Fluorescence microscopy with mCherry-GFP-LC3 plasmid was applied to study the autophagy flux, and cytoprotective effects were investigated with colorimetric MTT and LDH assays. Resvega induced autophagy by showing increased autolysosome formation and autophagy flux, and the change in the p62 and LC3 protein levels further confirmed the fluorescent microscopy results. Moreover, Resvega provided a clear cytoprotection under proteasome inhibition. These findings highlight the potential of the nutraceuticals containing resveratrol, omega-3 fatty acids and other nutrients in the prevention of ARPE-19 cell damage.

## 1. Introduction

Age-related macular degeneration (AMD) is the leading cause of irreversible blindness among the elderly in Western countries. The number of patients suffering from AMD is expected to multiply over the coming decades. AMD has a significant impact on the lives of patients due to the impairment of sharp and color vision that disturbs daily life; for example, face recognition and reading [1]. The direct medical costs of AMD treatment amount to billions of dollars in the U.S. alone, and tens of millions of dollars in European countries, making it a financial burden for public health services [2]. AMD can be divided into dry and wet forms with ~80% and ~20% prevalence, respectively. Currently, there are no effective treatments for dry AMD. The wet form of AMD can be treated with anti-vascular endothelial growth factor (anti-VEGF) injections, but this treatment only delays the progression of the disease. Therefore, there is a need for new preventive and medicinal approaches and therapies targeted against AMD.

AMD is characterized as progressive loss of central vision with degenerative and neovascular changes in the retina [1]. The etiology of AMD is multifactorial and includes genetic susceptibility, smoking, obesity, arteriosclerosis, hypertension, and unhealthy diet [3]. The cellular pathology of AMD coincides with oxidative stress, inflammation and protein aggregation [4,5]. The post-mitotic retinal pigment epithelial (RPE) cells are responsible for the maintenance of the retina by uptaking nutrients and removal of waste material, such as the outer segments of photoreceptor cells responsible for visual perception [6]. The role of RPEs as a “janitor” of the retina includes the maintenance of protein homeostasis by various mechanisms including two proteolytic pathways: the ubiquitin-proteasome system (UPS) and the lysosomal/autophagosomal degradation system (autophagy) [7]. The UPS is responsible for the degradation of 80%–90% of proteins, mostly short-lived or damaged proteins [8]. Autophagy is specialized to degrade aggregated proteins and cell organelles. Inefficient protein homeostasis affects the capability of the cell to tolerate stress, and *vice versa*. This can result in detrimental accumulation of lipid and protein aggregates, lipofuscin and drusen, which is a hallmark of AMD [9,10]. The number of aggregated proteins is increased by oxidative stress, making the autophagosomal degradation pathway greatly important [11]. Oxidative stress is known to be high in the macular area of the retina and, when chronic, it can overwhelm the RPE cells and cause loss of RPE and photoreceptor cells—the initial step of AMD pathology.

In autophagy, the ubiquitin-binding p62/SQSTM1 (p62) protein serves as a marker for the cargo that is subjected to autophagosomal degradation. The protein level of p62 may increase shortly after autophagy activation, but its content decreases over time in the autophagic degradation process along with the cargo [12]. The p62-marked cargo is then engulfed by the double-membrane organelle—the autophagosome—guided by p62-LC3 (microtubule-associated protein 1A/1B-light chain 3) interaction [13]. The LC3 protein has two forms, LC3-I and LC3-II: the LC3-I form is found in pre-autophagosomal structures and is converted to the LC3-II form in the autophagosome maturation process by phosphatidylethanolamine (PE) conjugation. After the cargo is isolated in the autophagosome, a lysosome is fused to form an autolysosome. Finally, the cargo and the inner membrane of the autophagosome and the lysosome are degraded by lysosomal hydrolytic enzymes [14].

The use and research of naturally-derived compounds for preventing and treating age-related diseases associated with autophagy and oxidative stress has grown over the past decade [15,16,17,18]. Growing evidence of stilbene compound’s ability to decrease oxidative stress [19,20] and ameliorate oxidative stress-related diseases [21] has drawn attention to autophagy research. Current data indicate that the key regulator of the oxidative stress defense system, nuclear factor-erythroid 2-related factor-2 (Nrf2) interacts closely with the autophagy process via the p62 protein [22]. Furthermore, the key regulator of autophagy, AMP-activated protein kinase (AMPK), has been shown to induce Nrf2-mediated protection against oxidative stress [23]. It is possible that stilbene compounds have a dual role in oxidative stress related diseases, activation of the antioxidant defense system, and autophagy.

Recently, resveratrol, a natural polyphenolic compound found mainly in grapes, peanuts, and berries, based on an oral supplement produced long-term beneficial effects on visual function in human patients [24]. Furthermore, resveratrol protected RPE cell lines from UV-induced oxidative stress [25] and suppressed VEGF expression in human RPE cells and, together with omega-3 fatty acids, in a mouse model of choroidal neovascularization [26,27], prevented the development of AMD. Docosahexaenoic acid (DHA), which is an omega-3 polyunsaturated fatty acid, has shown to induce autophagy in human retinal pigment epithelial cells (ARPE-19) and human cancer cells [28,29]. The Age-Related Eye Disease Study (AREDS) [30,31] demonstrated that the combination of an oral supplement consisting of antioxidant vitamins C (500 mg), E (400 international units), and β-carotene (15 mg), minerals (zinc (60 mg of zinc oxide) with copper (2 mg cupric oxide)) reduced the risk of intermediate AMD developing to advanced AMD by 25%. Furthermore, the risk of moderate vision loss was decreased by 19% in a five-year follow-up.

In this article, a commercial supplement (Resvega, formulated by Laboratoires Théa using the results from the AREDS study) was given to RPE cell lines; protein levels of p62, LC3-I/LC3-II, cytoprotective effects, and the number of autolysosomes were investigated to determine the activation of autophagy.

## 2. Materials and Methods

### 2.1. Cell Culture

The ARPE-19 cell line from American Type Culture Collection (ATCC, Manassas, VA, USA) was used in the experiments. The cells were grown in Dulbecco’s modified eagle medium/nutrition mix F12 (1:1 Gibco, Paisley, UK) with 10% fetal bovine serum (FBS, Hyclone, Logan, UT, USA), 100 µg/mL streptomycin (Lonza, Walkersville, MD, USA), 100 U/mL penicillin (Lonza) and 2 mM L-glutamine (Lonza) unless otherwise stated. The cells were grown at 37 °C in a humidified 5% CO_2_ atmosphere. Cells with passage numbers under 15 were being used.

### 2.2. Treatments

To study the effects of Resvega (Laboratoires Théa, Clermont-Ferrand, France) to induce autophagy, ARPE-19 cells were incubated in medium containing 288 ng Resvega corresponding to 25 µM of resveratrol (contents: 30 mg of trans resveratrol as a daily dose, 240 mg of vitamin C, 30 mg of vitamin E, 12.5 mg of zinc, 1 mg of copper, 665 mg of omega-3 fatty acids, 10 mg of lutein, and 2 mg of zeaxanthin), 1 µM proteasome inhibitor MG-132 (Calbiochem, Billerica, MA, USA), and 50 nM autophagy inhibitor bafilomycin A1 (Sigma-Aldrich, St. Louis, MO, USA). Approximately 250,000 cells/well were seeded in 12-well plates and incubated for 48 h to reach confluency. The old medium was removed and fresh medium containing Resvega, MG-132, bafilomycin A1, or their combinations diluted in dimethyl sulfoxide (DMSO) was added to the cell culture for 6–48 h. Control samples were treated with the same amount of DMSO found in the Resvega treatment samples (0.35% *v*/*v*). The treatments were also conducted without serum to study Resvega’s ability to increase starvation-induced autophagy in the ARPE-19 cell line. The serum-free cells were washed once with serum-free medium before treatment and another dose of compounds was added by changing fresh serum-free medium after 24 h of incubation to achieve cytotoxic effects by proteasome inhibition. Otherwise, the cells were treated as described above.

### 2.3. Western Blot Analyses of Autophagy Markers p62 and LC3-I/LC3-II

ARPE-19 cells were lysed with Mammalian Protein Extraction Reagent (M-PER, Thermo scientific, Rockford, IL, USA). Cells were washed twice with PBS (Dulbecco’s phosphate-buffered saline, Sigma-Aldrich) and then 75 µL of M-PER solution was added to each well for 3 min. The wells were scraped on ice and the lysates were collected and centrifuged at 13,000× *g* for 5 min at 4 °C. The protein content was measured using a Bradford protein assay from the supernatant. 20 µg of proteins were separated on 15% SDS-PAGE and transferred to a nitrocellulose membrane (Amersham Protran premium 0.45 µm NC, GE Healthcare, Germany). The membranes were blocked with 3% skim milk in 0.3% Tween PBS for 1.5 h. p62 primary antibody (sc-28359, Santa Cruz Biotechnology Inc., Santa Cruz, CA, USA) was diluted 1:1000 with 0.5% bovine serum albumin (BSA) in 0.3% Tween PBS and incubated over night at 4 °C. LC3-I/LC3-II primary antibody (#3868, Cell Signaling Technology, Danvers, MA, USA) was diluted 1:1000 with 5% BSA in 0.1% Tween Tris-buffered saline (TBS) and incubated over night at 4 °C, whereas alpha-Tubulin primary antibody (#T5168, Sigma-Aldrich, Saint Louis, MO, USA) was diluted 1:8000 with 1% skim milk in 0.05% Tween PBS for 15 min at room temperature. The secondary antibody for p62 and alpha-Tubulin was horseradish peroxidase (HRP)-linked anti-mouse (NA931, GE Healthcare) diluted 1:10,000 with 3% skim milk in 0.3% Tween PBS for 2 h at room temperature and 1% skim milk in 0.05% Tween PBS for 15 min at room temperature, respectively. The secondary antibody for LC3-I/LC3-II was HRP-linked anti-rabbit (Novex™, #A16014, ThermoFisher Scientific, Rockford, IL, USA) diluted 1:10,000 with 3% skim milk in 0.1 Tween TBS for 2 h at room temperature. The membranes were washed after primary and secondary antibody incubation with 0.3% Tween in PBS (p62), 0.1% Tween in TBS (LC3-I/LC3-II) or 0.05% Tween in PBS (alpha-Tubulin). After secondary incubation, the bands were visualized using Immobilon Western Chemiluminescent HRP Substrate (Millipore, Billerica, MA, USA) and an ImageQuant RT ECL system (GE Healthcare, Little Chalfont, UK). The band quantification was performed using ImageJ software (National Institutes of Health, Bethesda, MD, USA) and the band intensities of p62 and LC3-I/LC3-II were normalized against alpha-Tubulin.

### 2.4. Cell Viability Assays

Cell viability from serum-free samples were analyzed using a 3-(4,5 dimethylthiazol-2-yl)-2,5-diphenyltetrazolium bromide (MTT) assay [32] after 48 h of proteasome inhibition and/or Resvega incubation. The absorbance of lysate was measured in a spectrophotometer (Model 550 Microplate reader, BIO-RAD, USA) at 550 nm. The condition of the cells was assessed with a microscope before MTT assay to confirm the correlation of absorbance readings. Samples from the growth medium were taken for the lactate dehydrogenase (LDH) assay (CytoTox 96^®^ Non-Radioactive Cytotoxicity Assay, Promega, Madison, WI, USA) before the MTT-assay. 50 µL of growth medium from each sample was added to a 96-well flat bottom microtiter plate and 50 µL of LDH assay buffer was added and gently mixed. The plate was kept in the dark for 30 min before adding 50 µL of stop solution. After addition of the stop solution, the plate was gently mixed and absorbance was recorded at 490 nm.

### 2.5. Determination of Lysosome Fusion to the Autophagosome Using mCherry-GFP-LC3-Plasmid

Thirty-five thousand cells were plated to a Permanox eight-well chamber slide (#177445, Thermo Scientific Nunc^®^, Waltham, MA, USA) and incubated for 24 h. After reaching ~50%–60% confluency, cells were transfected with 500 ng/well of pH sensitive tandem mCherry-GFP-LC3-plasmid (kind gift from Drs Ana Maria Cuervo and Hiroshi Koga) using ExGen 500 *in vitro* transfection reagent (Fermentas, Burlington, ON, Canada). 24 h after transfection, cells were treated with 36 ng Resvega (corresponding the concentration used in the Western blot experiments) and/or MG-132 for 12 h. The control cells were treated with the same amount of DMSO found in the treatments. The cells were then fixed with 4% paraformaldehyde (PFA) for 15 min and the nuclei stained with DAPI 1:10,000 (4′,6-diamidino-2-phenylindole, Sigma-Aldrich) for 5 min. The cells were photographed using fluorescence microscopy (Axio Imager with ApoTome.2 (Carl Zeiss, Oberkochen, Germany) with Carl Zeiss ZEN imaging software) within 24 h. The samples were masked from the photographer. At neutral pH, both of the fluorophores fluoresce, indicating autophagosome presence (yellow), when a lysosome gets fused to an autophagosome during autolysosome maturation, the result is a lower pH and quenching of the GFP signal, which is seen as red fluorescence only (autolysosome). Autophagosome and autolysosome dots were then counted using ImageJ software.

### 2.6. Statistical Analyses

Data are presented as mean ± standard deviation (SD) (*n* = 3) for the Western blotting and cell viability experiments, and as mean ± standard error of the mean (SEM), (*n* = 9–12) for the fluorescence microscopy experiment. Statistical analyses were done using IBM SPSS Statistics software version 21 (IBM, Armonk, NY, USA). All of the data were subjected to one-way analysis of variance (ANOVA), followed by Tukey’s test for multiple comparison (*p* values < 0.05 were considered significant).

## 3. Results

### 3.1. Resvega Alters the Level of Autophagy Markers p62 and LC3

**Figure 1:**
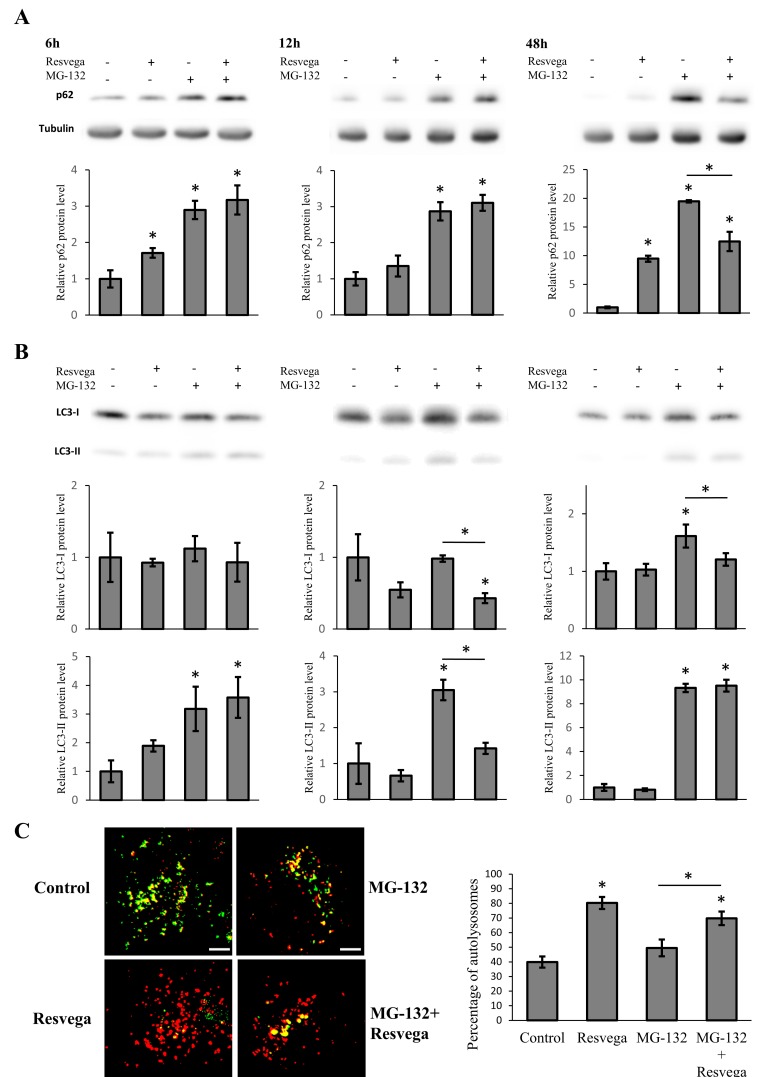
ARPE-19 cells were treated with 288 ng Resvega, 1 µM MG-132 or their combination for 6 h, 12 h, and 48 h in normal growth conditions. The protein level of p62 (**A**) and LC3-I/LC3-II (**B**) were analyzed by Western blot, while the expression was quantified in a comparison to α-tubulin and presented as fold change compared to control; and (**C**) cells were transfected with mCherry-GFP-LC3-plasmid and treated for 12 h, the fluorescence was then determined by fluorescence microscopy, red (autolysosomes) and yellow (autophagosomes) dots were counted and presented as the percentage of autolysosomes. Scale bar: 5 µm. Western blotting data are shown as mean ± SD (*n* = 3), and the percentage of autolysosomes is shown as mean ± SEM (*n* = 9–12). * *p* < 0.05, ANOVA.

ARPE-19 cells were treated with Resvega for 6 h, 12 h, and 48 h. Protein levels of autophagy markers p62 and LC3 were analyzed at each time point to study the activation of autophagy. Western blot data showed a mild increase in p62 content after Resvega treatment for 6 h and 12 h, the p62 level being significantly higher after Resvega treatment for 48 h (approximately nine-fold increase *vs.* control) (Figure 1A). The LC3-II protein level showed slight increase after 6 h of Resvega treatment, but decreased to baseline levels at 12 h and 48 h (Figure 1B). The protein expression of LC3-I was also decreased by Resvega treatment at 12 h, possibly due to the conversion of LC3-I to LC3-II in the autophagosome maturation (Figure 1B). The levels of LC3-I remained unchanged at 6 h and 48 h.

### 3.2. Resvega Faciliates Protein Clearance by Autophagy in Proteasome Inhibition Model

The ARPE-19 cells were treated with MG-132, a proteasome inhibitor, to create protein cargo for the autophagic machinery [33,34]. The MG-132 treatment significantly increased the protein level of p62 at all time points as expected, whereas simultaneous treatment with Resvega and MG-132 showed ~33% lower p62 content after 48 h (Figure 1A). The protein level of LC3-II was significantly increased by MG-132 treatment at all time points (Figure 1B), while simultaneous treatment with Resvega and MG-132 resulted in significantly lower LC3-II expression at 12 h. MG-132 treatment increased the LC3-I protein levels at 48 h, while at other time points, the LC3-I protein levels resembled the results obtained with Resvega alone (Figure 1B).

### 3.3. Resvega Induces Autophagic Flux by Increasing the Number of Autolysosomes

ARPE-19 cells were transfected with pH-sensitive mCherry-GFP-LC3-plasmid to monitor autophagic flux [35]. The cells were then treated with Resvega and/or MG-132 with the mCherry-GFP-LC3-plasmid for 12 h and analyzed with fluorescence microscopy to investigate autolysosome formation. Resvega treatment clearly elevated the portion of autolysosomes compared to control (Figure 1C). MG-132 treatment appeared to slightly elevate the portion of autolysosomes, whereas treatment with Resvega and MG-132 together elevated the portion of autolysosomes significantly when compared to MG-132 treatment. The observed changes in protein levels, such as a decrease of LC3-II at 12 h, support the fluorescence microscopy findings.

### 3.4. Resvega Enhances Autophagy and Increases Cell Viability in the Starvation-Induced Autophagy Model

**Figure 2:**
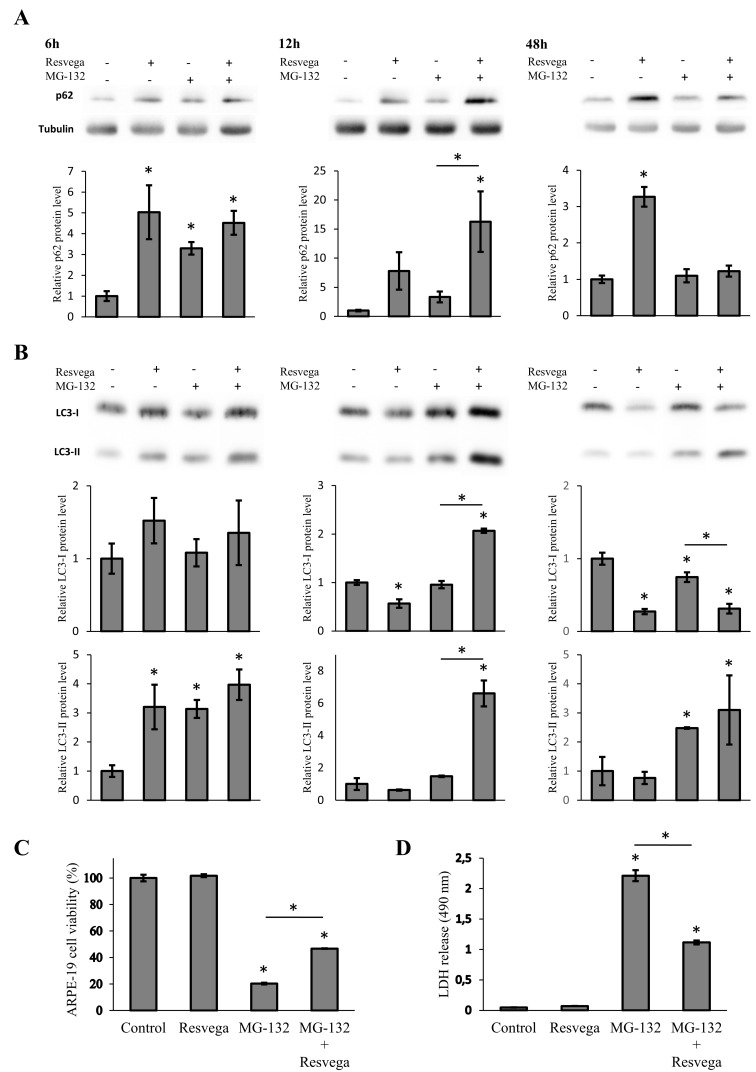
ARPE-19 cells were treated with 288 ng of Resvega, 1 µM MG-132 or their combination for 6 h, 12 h, and 48 h under serum starvation. The protein levels of p62 (**A**) and LC3-I/LC3-II (**B**) were analyzed by Western blot, while the expression was quantified in comparison to α-tubulin and presented as a fold change compared to control; the cells were treated for 48 h and MTT (**C**) and LDH (**D**) assays were performed. Western blot data are shown as mean ± SD (*n* = 3), MTT assay data as mean percentage (control = 100%) ± SD (*n* = 3) and LDH assay data as mean absorbance ± SD (*n* = 3). * *p* < 0.05, ANOVA.

Serum starvation increases autophagy when the cell recycles necessary nutrients that are normally provided in serum. ARPE-19 cells were treated in a starvation-induced autophagy model with Resvega and/or MG-132 proteasome inhibitor for 6 h, 12 h, and 48 h. Protein levels of p62 and LC3 were analyzed by Western blotting. When autophagy was already heavily activated by serum starvation, Resvega and/or MG-132 elevated the expression of p62 significantly at 6 h and 12 h, whereas Resvega treatment alone maintained high p62 levels at 48 h (Figure 2A). Resvega, together with MG-132, increased p62 content more than MG-132 alone at 6 h and 12 h. The protein levels of LC3-II were elevated by Resvega and/or MG-132 treatment at 6 h, however, at 12 h the LC3-II content decreased back to the baseline level and was below the baseline level obtained with Resvega (Figure 2B). Only the combined treatment of Resvega and MG-132 maintained high LC3-II levels after 12 h of treatment. At 48 h, MG-132 alone and Resvega + MG-132 both showed elevated levels of LC3-II. The highest LC3-II content was found with Resvega + MG-132. The levels of LC3-I were significantly higher with Resvega + MG-132 treatment at 12 h, whereas lower levels of LC3-I were observed with Resvega alone (Figure 2B). After 48 h of treatment, Resvega alone and Resvega + MG-132 clearly showed decreased levels of LC3-I. The decrease of LC3-I together with the increase of LC3-II can be explained by the conversion of LC3-I to LC3-II when autophagy is induced.

The LDH release, a biomarker for cytotoxicity and cytolysis, and MTT assay measuring the function of mitochondria were performed for the serum starvation-induced autophagy model to monitor toxic effect of protein accumulation via proteasome inhibition by MG-132. These protein aggregates can be removed via autophagosomal degradation and the possible alleviation of this stress can be monitored with cytotoxicity assays. Resvega itself did not increase the levels of LDH release nor decreased cell viability in the MTT assay; MG-132 showed clear cytotoxic effects with both methods after 48 h of treatment (Figure 2C,D). Resvega was able to alleviate the cytotoxic effects of MG-132, possibly by inducing autophagy.

### 3.5. Resvega Induces Accumulation of LC3 and p62 under Autophagy Inhibition

**Figure 3:**
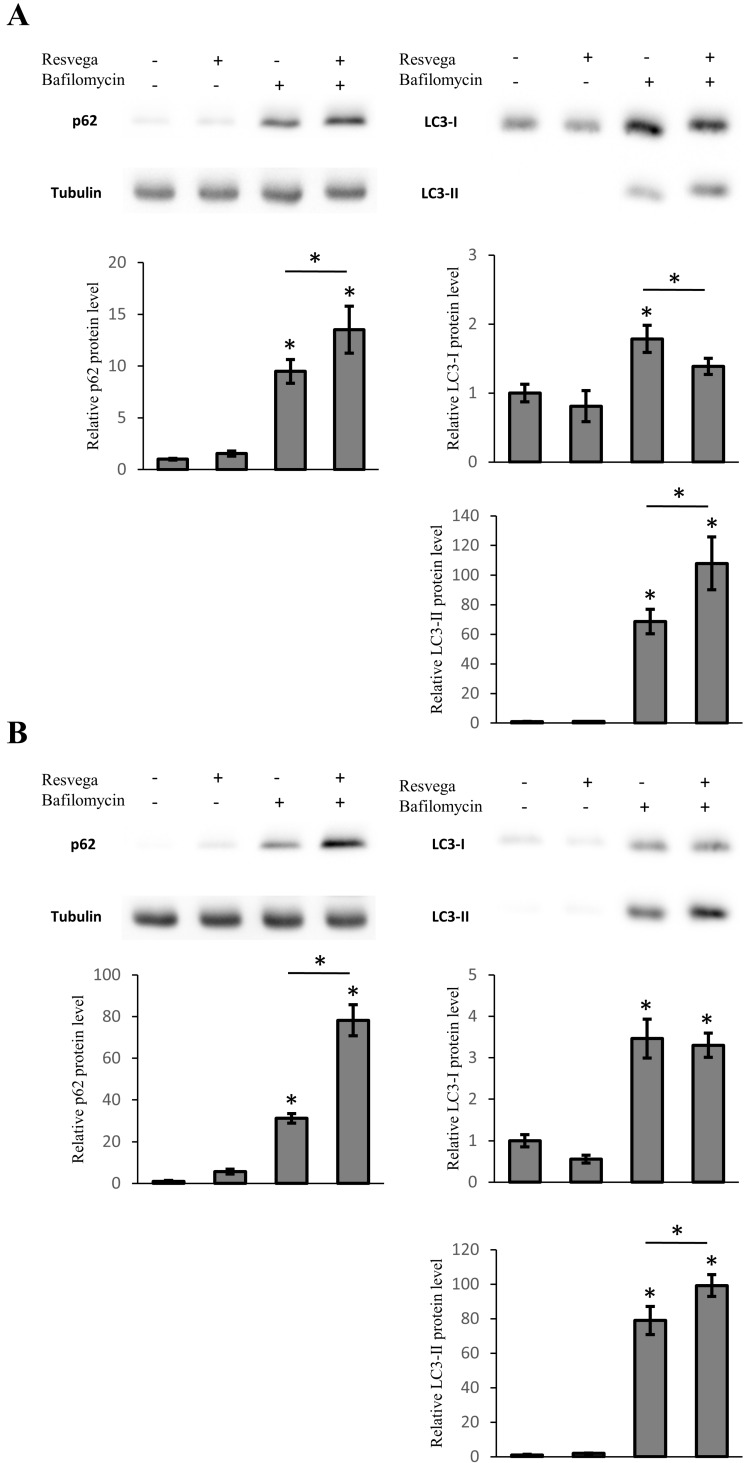
ARPE-19 cells were treated with 288 ng Resvega, 50 nM bafilomycin A1, or their combination for 12 h (**A**) in normal growth conditions and (**B**) a starvation-induced autophagy model. The protein level of p62 and LC3-I/LC3-II were analyzed by Western blot, while the expression was quantified in a comparison to α-tubulin and presented as a fold change compared to control. Western blotting data are shown as mean ± SD (*n* = 3). * *p* < 0.05, ANOVA.

ARPE-19 cells were treated with bafilomycin A1, an inhibitor of vacuolar type H(+)-ATPase, preventing the acidification of lysosomes and formation of autolysosomes, for 6 h and 12 h to investigate effects of Resvega on the accumulation of LC3-II or p62. The bafilomycin treatment significantly increased the protein levels of LC3-II and p62 at both time points as a result of autophagy inhibition (Figure 3 and supplementary materials Figure S1), whereas simultaneous treatment with Resvega and bafilomycin showed significant accumulation of LC3-II and p62 in a starvation-induced autophagy model at 6 h (supplementary materials Figure S1). After 12 h of treatment, the protein levels of LC3-II and p62 also increased significantly in normal growth conditions (Figure 3A). The accumulation of LC3-II and p62 continued in the starvation-induced autophagy model at 12 h (Figure 3B). Interestingly, the amount of LC3-I was decreased and the LC3-II was increased at 12 h in normal growth conditions that resemble the results obtained with the proteasome inhibitor MG-132 (Figure 1B). The accumulation rate of LC3-II and p62 are in correlation with the results obtained by Resvega alone when comparing normal growth conditions and the starvation-induced autophagy model (Figure 1 and Figure 2).

## 4. Discussion

In this study, Resvega was able to activate autophagic machinery and further increase autophagy in the starvation model. Moreover, Resvega contributed to the survival of ARPE-19 cells exposed to detrimental protein waste triggered by proteasome inhibition.

The p62 protein is the key regulator of autophagy, binding to protein aggregates and guiding them into autophagosomal degradation. Macula samples from human AMD patients show accumulation of p62, suggesting declined autophagosomal degradation and highlighting the importance of autophagy in AMD [33]. Classically, during autophagy, the p62 level is expected to decrease due to degradation during the autophagic process, as shown with resveratrol [36,37]. However, in our study, the level of p62 increased under Resvega treatment after 6 h and continued to increase during the monitored time period. Resvega contains omega-3 fatty acids, and marine omega-3 polyunsaturated fatty acids that have been shown to increase the amount of p62 in ARPE-19 cell lines, which might enhance the selectivity of damaged or aggregated proteins towards autophagy [28,38]. Moreover, the increase in the p62 protein level with Resvega might be explained by the lack of protein substrate for autophagy, whereas proteasome inhibition with MG-132 provides the necessary protein substrate and explains the decrease of p62. The behavior of p62 was similar in the starvation model with the exception that treatments, especially with Resvega, had a greater effect when autophagy was activated. Interestingly, p62 levels returned to baseline after proteasome inhibition for 48 h, indicating active removal of protein waste.

Resvega significantly increased the level of LC3-II after 6 h of treatment indicating an enhancement of autophagosome formation and packaging of waste material for autophagic degradation. However, the level of LC3-II had decreased to below the baseline level after 12 h of treatment. This could be another sign of autophagy activation since autophagosomes, with LC3-II in the membrane structures, are degraded during the autophagy process [13]. Similar results with LC3-II levels using resveratrol have been seen with human dermal fibroblasts and human umbilical vein endothelial cells [39,40]. The LC3-II levels were also significantly increased by the proteasome inhibitor MG-132, indicating autophagosome formation. However, only co-treatment with Resvega led to decreased LC3-II levels at 12 h, probably due to further enhanced autophagic flux. The level of LC3-I supports this theory since the LC3-I level was significantly decreased at the same time point, possibly due to conversion of LC3-I to LC3-II in the autophagosome maturation process. In the starvation model, the LC3-II levels were increased by Resvega, MG-132, and their combination after 6 h of treatment. Six hours later, only co-treatment maintained high levels of LC3-II, suggesting further induced autophagosome formation by Resvega. Interestingly, the levels of LC3-II were also high at 48 h, despite normal p62 levels. Apparently, cells were able to remove excess proteins by autophagy, resulting in increased LC3-II levels, yet maintaining baseline p62 levels. Another interesting result is the level of LC3-I, which was decreased significantly by co-treatment than by MG-132 alone, at 48 h. This may indicate better LC3-II conversion and, perhaps, more efficient autophagy. Autophagy inhibition with bafilomycin showed high accumulation of both LC3-II and p62 in Resvega treated samples implicating Resvega’s ability to induce autophagy without the presence of proteasome inhibition or any other treatment such as starvation.

The use of mCherry-GFP-LC3 plasmid for monitoring the number autophagosomes and autolysosomes further confirmed the results observed from Western blotting. The percentage of lysosomes in Resvega treated samples was significantly higher compared to control and MG-132 samples. However, the proteasome inhibition increased not only the number of autolysosomes but also the number of autophagosomes (data not shown). This could be a result of inefficient autophagic flux where autophagic machinery is overwhelmed with waste proteins caused by proteasome inhibition. As a result, the rate of autophagic flux is not efficient and autophagosomes start to accumulate. Co-treatment with Resvega facilitated the autophagic flux and accumulation of autophagosomes was, therefore, not observed.

LDH and MTT cell viability tests confirmed the capability of Resvega to protect ARPE-19 cells from harmful protein caused by proteasome inhibition. Similar results have been observed in human neuroblastoma cells where resveratrol protected cells from rotenone-induced cell death by activating autophagy and the antioxidant defense system [36]. In this experiment, the p62 and LC3-II levels of MG-132 and MG-132 + Resvega samples were equal when cell viability tests were performed. However, there was a clear difference in LDH and MTT assay data between MG-132 and MG-132 + Resvega samples. In addition to the activation of autophagy, this could be explained by the concurrent activation of antioxidant defense mechanisms [28]. It has been shown that p62 is closely involved in the regulation of the Nrf2/ARE antioxidant defense pathway [22,41]. Moreover, resveratrol, which is present in Resvega, can directly activate the Nrf2/ARE pathway [36]. When autophagy was inhibited with bafilomycin, the accumulation of both p62 and LC3-II suggests that Resvega has a dual role as an autophagy and Nrf2/ARE activator.

Resvega, a commercial supplement developed following the AREDS study, clearly activated autophagy by modulating p62 and LC3 levels and by increasing the number of autolysosomes in ARPE-19 cells. However, other models, such as stem cells, primary cells, and human materials are required to support these findings. As such, Resvega seems to be a promising supplement for preventing and treating age-related diseases associated with impaired autophagy.

## Supplementary Materials

The following are available online at http://www.mdpi.com/2072-6643/8/5/284/s1, Figure S1, ARPE-19 cells were treated with 288 ng Resvega, 50 nM bafilomycin A1 or their combination for 6 h (A) in normal growth conditions and (B) starvation-induced autophagy model. The protein level of p62 and LC3-I/LC3-II were analyzed by Western blot, while the expression was quantified in a comparison to α-tubulin and presented as fold change compared to control. Western blotting data are shown as mean ± SD (*n* = 3). * *p* < 0.05, ANOVA.
