# Supplementary material for: Nutraceutical with Resveratrol and Omega-3 Fatty Acids Induces Autophagy in ARPE-19 Cells
Source: Nutrients. 2016 May 11;8(5):284. doi: 10.3390/nu8050284 (PMC4882697; doi:10.3390/nu8050284)
Supplement: Supplementary file 1 [file nutrients-08-00284-s001.docx]

Supplementary Materials: Nutraceutical with Resveratrol and Omega-3 Fatty Acids Induces Autophagy in ARPE-19 Cells

Ali Koskela, Mika Reinisalo, Goran Petrovski, Debasish Sinha, Céline Olmiere,
Reijo Karjalainen and Kai Kaarniranta


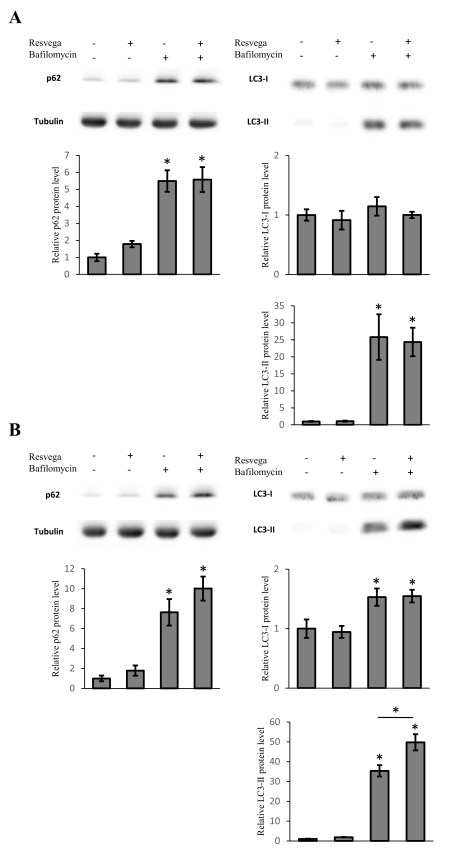


**Figure S1**. ARPE-19 cells were treated with 288 ng Resvega, 50 nM bafilomycin A1 or their combination for 6 h (**A**) in normal growth conditions and (**B**) the starvation-induced autophagy model. The protein level of p62 and LC3-I/LC3-II were analyzed by Western blot, while the expression was quantified in a comparison to α-tubulin and presented as a fold change compared to control. Western blotting data are shown as mean ± SD (*n* = 3). * *p* < 0.05, ANOVA.
